# Trajectory of patients over five years in Multispecialty Interprofessional Team Memory Clinics

**DOI:** 10.1002/alz.087436

**Published:** 2025-01-03

**Authors:** Aaron Jones, Linda Lee, Tejal Patel

**Affiliations:** ^1^ McMaster University, Hamilton, ON Canada; ^2^ McMaster University, Kitchener, ON Canada; ^3^ MINT Memory Clinic, Kitchener, ON Canada; ^4^ Schlegel‐UW Research Institute on Aging, Waterloo, ON Canada; ^5^ University of Waterloo, Waterloo, ON Canada

## Abstract

**Background:**

Multispecialty Interprofessional Team (MINT) Memory Clinics manage dementia care in primary care, allowing for more efficient use of limited specialist resources. This study examined the characteristics of patients on their initial assessment in the MINT clinic and investigated the five‐year trajectory of patients with mild cognitive impairment (MCI).

**Method:**

We conducted a retrospective chart review of 751 patients assessed within a MINT Memory Clinic between June 2006 and May 2019 to collect data on age, gender, diagnosis, and MoCA scores. We plotted the five‐year trajectory of MoCA scores for patients with an initial diagnosis of MCI and calculated product‐limit estimates of the five‐year conversion rate to dementia. We also performed a Cox regression to investigate associations with receiving a dementia diagnosis.

**Result:**

The median age of our cohort was 75 years; 54% were female. The majority of patients (70%) had a MoCA score of 25 or less; 35% had a score of 20 or less. Diagnoses at the initial assessment included MCI (37%), dementia (20%), subjective cognitive decline (SCD; 12%), and other miscellaneous diagnoses (3%); 27% did not a receive a definitive diagnosis, pending further investigations. Of the 214 patients with an initial diagnosis of MCI and at least one follow‐up visit, 28% (N = 60) received a dementia diagnosis at a later date, with 58% being diagnosed with mixed dementia, 22% with Alzheimer’s Disease, and 20% with other dementias. The product‐limit estimates of the five‐year conversion rate was 48.8% (39.5%, 59.2%). Lower MoCA scores at the initial visit were associated with a higher risk of a dementia diagnosis; each one‐point increase in MoCA was associated with a 10% decrease in the risk of a dementia diagnosis (aHR: 0.90, 95%CI: 0.85‐0.96). Similarly, age was significantly associated with a higher risk of a dementia diagnosis within five years; each five‐year increase in age was associated with a 37% increase in risk (aHR: 1.37, 95% CI: 1.12‐1.67).

**Conclusion:**

As nearly half of the patients presenting to MINT Memory Clinics have MCI or SCD on initial assessment, MINT clinics provide a significant opportunity for detection and potential intervention at early disease stages.

